# PDK1-Foxo1 in Agouti-Related Peptide Neurons Regulates Energy Homeostasis by Modulating Food Intake and Energy Expenditure

**DOI:** 10.1371/journal.pone.0018324

**Published:** 2011-04-07

**Authors:** Yongheng Cao, Masanori Nakata, Shiki Okamoto, Eisuke Takano, Toshihiko Yada, Yasuhiko Minokoshi, Yukio Hirata, Kazunori Nakajima, Kristy Iskandar, Yoshitake Hayashi, Wataru Ogawa, Gregory S. Barsh, Hiroshi Hosoda, Kenji Kangawa, Hiroshi Itoh, Tetsuo Noda, Masato Kasuga, Jun Nakae

**Affiliations:** 1 Division of Molecular Medicine and Medical Genetics, International Center for Medical Research and Treatment (ICMRT), Kobe University Graduate School of Medicine, Kobe, Japan; 2 21st Century COE Program for Signal Transduction Disease: Diabetes Mellitus as Model, Division of Diabetes, Metabolism, and Endocrinology, Department of Internal Medicine, Kobe University Graduate School of Medicine, Kobe, Japan; 3 Division of Integrative Physiology, Department of Physiology, School of Medicine, Jichi Medical University, Tochigi, Japan; 4 Division of Endocrinology and Metabolism, Department of Developmental Physiology, National Institute for Physiological Sciences, Aichi, Japan; 5 Department of Anatomy, Keio University School of Medicine, Tokyo, Japan; 6 Division of Diabetes, Metabolism, and Endocrinology, Department of Internal Medicine, Kobe University Graduate School of Medicine, Kobe, Japan; 7 Departments of Genetics and Pediatrics, Stanford University, Stanford, California, United States of America; 8 Department of Biochemistry, National Cardiovascular Center Research Institute, Fujishirodai, Suita, Japan; 9 Frontier Medicine on Metabolic Syndrome, Division of Endocrinology, Metabolism and Nephrology, Department of Internal Medicine, Keio University School of Medicine, Tokyo, Japan; 10 Department of Cell Biology, Japanese Foundation for Cancer Research, Cancer Institute, Tokyo, Japan; 11 Research Institute, International Medical Center of Japan, Tokyo, Japan; University of Las Palmas de Gran Canaria, Spain

## Abstract

Insulin and leptin intracellular signaling pathways converge and act synergistically on the hypothalamic phosphatidylinositol-3-OH kinase/3-phosphoinositide-dependent protein kinase 1 (PDK1). However, little is known about whether PDK1 in agouti-related peptide (AGRP) neurons contributes to energy homeostasis. We generated AGRP neuron-specific PDK1 knockout (*AGRPPdk1^−/−^*) mice and mice with selective expression of transactivation-defective Foxo1 (*Δ256Foxo1^AGRP^Pdk1^−/−^*). The *AGRPPdk1^−/−^* mice showed reductions in food intake, body length, and body weight. The *Δ256Foxo1^AGRP^Pdk1^−/−^* mice showed increased body weight, food intake, and reduced locomotor activity. After four weeks of calorie-restricted feeding, oxygen consumption and locomotor activity were elevated in *AGRPPdk1^−/−^* mice and reduced in *Δ256Foxo1^AGRP^Pdk1^−/−^* mice. *In vitro*, ghrelin-induced changes in [Ca^2+^]_i_ and inhibition of ghrelin by leptin were significantly attenuated in *AGRPPdk1^−/−^* neurons compared to control neurons. However, ghrelin-induced [Ca^2+^]_i_ changes and leptin inhibition were restored in *Δ256Foxo1^AGRP^Pdk1^−/−^* mice. These results suggested that PDK1 and Foxo1 signaling pathways play important roles in the control of energy homeostasis through AGRP-independent mechanisms.

## Introduction

Obesity occurs when caloric intake exceeds ongoing energy requirements. Although environmental and lifestyle factors contribute to obesity, it has been proposed that homeostatic regulators control energy balance. The hypothalamus receives and integrates neural, metabolic, and humoral signals from the periphery. The two best-characterized populations of neurons in the brain reside within the hypothalamic arcuate nucleus, the anorexic pro-opiomelanocortin (POMC) neurons, which express POMC and cocaine and amphetamine-related transcript (CART) and the orexigenic agouti-related peptide (AGRP) neurons, which express AGRP and neuropeptide Y (NPY) [1]. Insulin and leptin mediate signals in the hypothalamic arcuate nucleus that inhibit food intake [2], [3].

Activated insulin receptors phosphorylate the insulin receptor substrate (IRS) proteins, which then activate phosphatidylinositol-3 kinase (PI(3)K); this generates phosphatidylinositol-3, 4, 5-trisphosphate (PIP3) and phosphatidylinositol-4, 5-bisphosphate (PIP2). PIP3 activates phosphatidylinositol-3-OH kinase/3-phosphoinositide-dependent protein kinase 1 (PDK1), which activates protein kinase B (PKB, also known as Akt) and members of the atypical PKC family. Among the targets of activated PKB are the mammalian target of rapamycin (mTOR) and the forkhead box transcription factor Foxo1 [1].

Leptin is a key starvation factor. During starvation, a reduction in leptin alters the tone of a large number of behavioral, endocrine, and autonomic circuits; these trigger an increase in food intake and a reduction in energy expenditure to restore energy homeostasis [4]. The activated leptin receptor stimulates Janus kinase-2 (JAK2) to phosphorylate signal transducer and activator of transcription-3 (STAT3). Phosphorylated STAT3 dimerizes and enters the nucleus to regulate the transcription of target genes. Leptin also activates the IRS-PI(3)K pathway [1], therefore, both insulin and leptin signaling pathways converge at PI(3)K-PDK1.

The PDK1-Foxo1 pathway in POMC neurons has an important role in inhibition of *Pomc* gene expression and regulates food intake [5], [6]. However, little is known about the role of PDK1 in controlling energy homeostasis in AGRP neurons. In the present study, we generated and analyzed AGRP neuron-specific *Pdk1* knockout mice to examine the roles of PDK1 in energy homeostasis. Furthermore, to investigate whether Foxo1 acts downstream of PDK1 in AGRP neurons, we generated *Pdk1* knockout mice that expressed an AGRP neuron-specific, transactivation-defective form of Foxo1 (Δ256Foxo1), which lacks a carboxyl terminal transactivation domain and can compete with endogenous Foxos and inhibit expression of the target genes of Foxos [6], [7], [8].

## Methods

### Mice

All experimental protocols with mice were approved by the animal ethics committees of the Keio University School of Medicine (#09134-(1)) and Kobe University Graduate School of Medicine (P-041004).

#### AGRP neuron-specific PDK1 knockout mice (AGRPPdk1^−/−^)

*Pdk1^flox/flox^* mice harbored an endogenous *Pdk1* gene with *loxP* sites flanking exons 3 and 4 [9]. These mice were bred with mice that expressed the *Cre* recombinase gene under the control of the murine *Agrp* promoter [10]. *Pdk1^flox/flox^* mice were genotyped by polymerase chain reaction (PCR) amplification of genomic DNA isolated from tail tips using the primers: 5′- GCCTCTTTAGGCTTTTGGAGTCGGC -3′ and 5′- GGAGAGGAGGAATGTGGACAAACAGC -3′.

#### AGRP neuron-specific Δ256Foxo1-transgenic mice (Δ256Foxo1^AgRP^Pdk1^−/−^)

The generation of *R26^floxneoΔ256Foxo1^* mice was described previously [6]. Breeding colonies were maintained by mating *R26^floxneoΔ256Foxo1^* mice with *AGRPCre* mice. Only animals from the same generation of the mixed-background strain were compared. Mice were genotyped by PCR analysis of genomic DNA isolated from the tail tips. *R26^floxneoΔ256Foxo1^* mice were identified with the primers: 5′-ATGGACTACAAAGACGATGAC-3′ and 5′-GTCGAGTTGGACTGGTTAAAC-3′. *AGRPCre* mice were identified with the primers: 5′-CCGCAGAACCTGAAGATGTTCGC-3′ and 5′-CAGATTACGTATATCCTGGCAGCG-3′. All mice studied were examined on a B6/129 mixed genetic background.

### Standard animal procedures and measurements

Animals were maintained in sterile cages in a barrier animal facility with a 12/12-h light/dark cycle. A standard chow diet and water were provided ad libitum unless otherwise specified. All assays were performed in duplicate. Values are reported as the mean of two independent determinations. We measured glucose levels with a Glutest Pro (Sanwa Kagaku Kenkyusho Co., Japan), insulin and leptin by sensitive rat insulin RIA kit (SRI-13K, Millipore, Billerica, MA) and mouse leptin RIA kit (ML-82K, Millipore, Billerica, MA). Glucose and insulin tolerance tests were performed as described previously [8]. In brief, for glucose tolerance test, we subjected mice to an overnight fast followed by intraperitoneal glucose injection (1.2 g/kg). We obtained blood samples at 0 15 30 60 and 120 min after the injection. For insulin tolerance test, we subjected mice to a 3-hour fast, followed by intraperitoneal insulin injection (1 U/kg). We obtained blood samples at 0, 20, 40, 60, 90 and 120 min and measured glucose levels. Measurements of serum leptin levels were performed as described elsewhere [8]. Serum thyroxine was measured with the Rodent T4 ELISA Test Kit (#ERK R7014, Endocrine Technology, Inc, Newark, CA). Serum corticosterone was measured with the Corticosterone Correlate-EIA™ Kit (#CM526791, Assay designs, Inc, Ann Arbor, MC). Serum norepinephrine was measured with the Norepinephrine ELISA Test Kit (#IB89537, IBL, Inc, Minneapolis, MN). CT scanning was performed using LaTheta LCT-100M (ALOKA CO., LTD, Japan).

### Calorie-restricted feeding studies

Male 20-week-old mice were housed individually. Food intake was monitored by weighing the chow every 24 h for 2 weeks. Then, each day, mice were given 70–80% of the daily food consumed by *AGRPPdk1^−/−^* mice because they consumed the least amount of food among control, *AGRPPdk1^−/−^*, and *Δ256Foxo1^AgRP^Pdk1^−/−^* mice. We confirmed that all mice consumed all the food provided everyday. After 4 weeks of Calorie-restricted feeding, we performed indirect calorimetry, recorded locomotor activity, conducted a leptin tolerance test, administered ghrelin, and administered melanotan II (MTII).

### Immunofluorescence

For immunofluorescence analyses, mice were transcardially perfused with saline followed by 4% paraformaldehyde in 0.1 M phosphate-buffered saline, pH 7.4 (PBS). The brains were dissected and immersed in 4% paraformaldehyde at 4°C overnight and then soaked in 30% sucrose overnight. Frozen, free-floating coronal sections (4 µm thick) were cut through the arcuate nucleus with a microtome (Leica Microsystems). The sections were washed extensively in PBS for 20 min to quench endogenous peroxidase activity. For double staining of PDK1 and AGRP, the sections were stained with a Renaissance Tyramide Signal Amplification kit (#NEL701, Perkin Elmer, Waltham, MA) according to the manufacturer's protocol. The primary antibodies were Ab-241 (Signalway Antibody, Pearland, TX) for PDK1 and GT15023 (Neuromics, Edina, MN) for AGRP; the secondary antibodies were Alexa Fluor^R^ 594 chicken anti-rabbit IgG and Alexa Fluor^R^ 488 donkey anti-goat IgG (Molecular Probes, Eugene, OR). For double staining of Foxo1 and AGRP, we used an anti-FOXO1A antibody (ab12161, abcam^R^, Cambridge, UK), respectively. For double staining of FLAG and AGRP, the sections were stained using a Renaissance Tyramide Signal Amplification kit according to the manufacturer's protocol. The primary antibodies were the OctA-Probe (D-8: sc-807, Santa Cruz Biotechnology, Inc, Santa Cruz, CA); the secondary antibodies were Alexa Fluor^R^ 594 chicken anti-rabbit IgG and Alexa Fluor^R^ 488 donkey anti-goat IgG (Invitrogen, Carlsbad, CA).

For quantification of PDK1, nuclear Foxo1, or FLAG expression in AGRP neurons, tissues were processed as described above for the double staining procedures. Pictures from every fourth section were taken throughout the arcuate nucleus (Bregma −1.1 mm to−2.7 mm), and all sections were positioned in the rostal to caudal direction to visualize the distribution of AGRP neurons. Neurons positive for both AGRP and PDK1, nuclear Foxo1, or FLAG immunoreactivity were counted and marked digitally to prevent multiple counts with Adobe Photoshop CS4 EXTENDED and ImageJ software (NIH; Bethesda, MD), as previously described [11]. Cell counts were performed in three mice per genotype. At least 150 cells were counted in each mouse.

### Preparation of single ARC neurons and measurement of [Ca^2+^]_i_

The preparation of single ARC neurons and measurement of cytosolic Ca^2+^ concentration ([Ca^2+^]_i_) were also described previously [6]. Briefly, after incubation with 2 µM fura-2/AM for 30 min at room temperature, cells were mounted in a chamber and superfused with HKRB containing 10 mM glucose at 1 ml/min at 33°C. The [Ca^2+^]_i_ was measured by ratiometric fura-2 fluorescence imaging with an intensified charge-coupled device camera. The ratio image was produced by an Aquacosmos system (Hamamatsu Photonics Co., Japan). Next, the cells were fixed with 4% paraformaldehyde for 30 min [12]. Immunocytochemical identification of AGRP neurons was performed with anti-AGRP antibody and the ABC method.

### RNA isolation and real-time PCR

Total RNA from each hypothalamus was isolated with the SV Total RNA Isolation System (#Z3100, Promega, Madison, WI) according to the manufacturer's protocol. The real-time PCR procedure [8] and primers [6] have been described previously.

### Leptin tolerance test

Male, 14–16 week-old, control and *AgRPPdk1^−/−^* mice were fed a normal chow diet. Body weight and food intake were measured for two days prior to the experiment. Mice were injected intraperitoneally with leptin (#450-31 Peprotech, Rocky Hill, NJ) twice a day (1.0 µg/g of body weight) for three days as described elsewhere [13]. Negative control mice were injected with the same volume of saline. Body weight and food intake were monitored for seven days.

### MTII and ghrelin responses

MTII (80 µg; H-3902, Bachem, Torrence, CA) or 100 µl saline was injected intraperitoneally, 30 min before lights out and food intake was monitored for 24 h [14]. Ghrelin (120 µg/kg) (#4373S, Peptide Institute, Japan) or 100 µl saline was injected intraperitoneally at 0900 h (in early light cycle) after a 24 h fast and food intake was monitored for 24 h.

### Oxygen consumption and physical activity

Six-month-old mice under normal chow diet were monitored individually in a metabolic cage (RL-600; Arco System, Kashiwa, Japan.) with free access to a normal chow diet and drinking water for 72 hours. Each cage was monitored for oxygen consumption at 5 minutes interval throughout 72 hours period. Total oxygen consumption was calculated as accumulated oxygen uptake for each mouse divided by its body weight. We performed oxygen consumption of ten mice in each genotype. Representative graphs were drawn from the mean and standard error calculated from data obtained in each measurement.

### Statistical analysis

We evaluated the significance of differences between groups by ANOVA followed by the Fisher's test (Statview; SAS Institute Inc.). P-values of less than 0.05 indicated statistical significance.

## Results

### Generation of *AGRPPdk1^−/−^* mice

**Figure 1:**
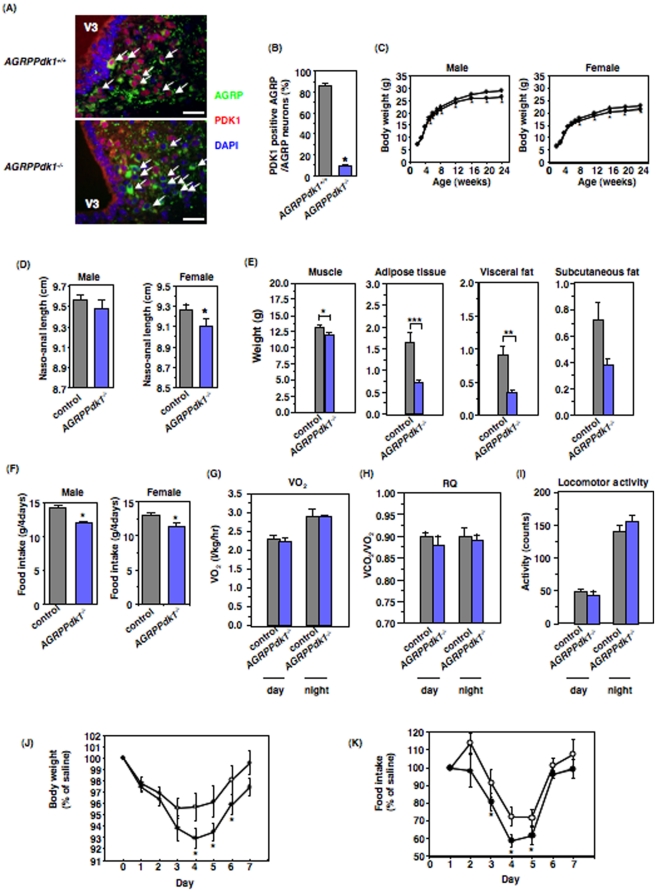
Generation and metabolic characterization of *AGRPPdk1^−/−^* mice. (**A**) Representative immunofluorescence images of hypothalamic regions doubly labeled for AGRP and PDK1 in 24-week-old *AGRPPdk1^+/+^* (top panel) and *AGRPPdk1^−/−^* (bottom panel) mice fed *ad libitum*. Green, red and blue indicate AGRP, PDK1, and DAPI staining, respectively. White arrows indicate AGRP neurons. Scale bars indicate 50 µm. (**B**) Quantification of PDK1-positive AGRP neurons from *AGRPPdk1^+/+^* (gray bar) and *AGRPPdk1^−/−^* (blue bar) mice. The percentage of PDK1-positive AGRP neurons among AGRP neurons was determined as described in Experimental Procedures. Values are means ± SEM of three mice of each genotype. An asterisk indicates a statistically significant difference between *AGRPPdk1^+/+^* and *AGRPPdk1^−/−^* mice with p<0.001 (one-factor ANOVA). (**C**) Body weight of male (left panel) and female (right panel) control (open circles) and *AGRPPdk1^−/−^* (closed circles) mice fed normal chow. Values represent mean ± SEM of 35–40 mice in each genotype. An asterisk indicates a statistically significant difference between control and *AGRPPdk1^−/−^* mice with p<0.05 (one-factor ANOVA). (**D**) Naso-anal length (NAL) of male (left panel) and female (right panel) control mice (gray bar) and *AGRPPdk1^−/−^* mice (blue bar) at 24 weeks of age. Values represent mean ± SEM of 30 mice in each genotype. An asterisk indicates a statistically significant difference between control and *AGRPPdk1^−/−^* mice with p<0.05 (one-factor ANOVA). (**E**) The calculated weights of muscle, adipose tissue, visceral fat, and subcutaneous fat in male control (gray bar) and *AGRPPdk1^−/−^*(blue bar) mice at 24 weeks of age. Skeletal muscle weight, adipose tissue weight (sum of visceral and subcutaneous fats), visceral fat weight, and subcutaneous fat weight were calculated as described in Experimental Procedures. The data represent the mean weight ± SEM of 10 mice per genotype. Asterisks indicate statistically significant differences between control and *AGRPPdk1^−/−^* mice with *p<0.001, **p<0.005, and ***p<0.05 (one-factor ANOVA). (**F**) Four-day food intake of male and female control mice (gray bar) and *AGRPPdk1^−/−^* mice (blue bar) at 24 weeks of age. Values represent mean ± SEM of 40 mice in each genotype. An asterisk indicates a statistically significant difference between control and *AGRPPdk1^−/−^* mice with p<0.05 (one-factor ANOVA). (**G**) Oxygen consumption in male control (gray bar) and *AGRPPdk1^−/−^*(blue bar) mice at 24 weeks of age. The values represent the mean ± SEM of 10 male mice per genotype. (**H**) Respiratory quotient of male control (gray bar) and *AGRPPdk1^−/−^*(blue bar) mice at 24 weeks of age. The values represent the mean ± SEM of 10 mice per genotype. (**I**) Basal locomotor activity for male control (gray bar) and *AGRPPdk1^−/−^*(blue bar) mice at 24 weeks of age during the light and dark phases. The values represent the mean ± SEM of 5 mice per genotype. (**J–K**) Effects of intraperitoneal leptin injections on body weight and food intake. Leptin was injected into control (open circle, n = 8) or *AGRPPdk1^−/−^* mice (closed circle, n = 5) for three consecutive days. Body weight (J) and food intake (K) were monitored for seven days. Body weight and food intake are presented as percentages of values from saline-injected mice for each genotype. An asterisk in (J) indicates a statistically significant difference between control and *AGRPPdk1^−/−^* mice with p<0.05 (one-factor ANOVA). An asterisk in (K) indicates a statistically significant difference between saline- and leptin-injected mice in *AGRPPdk1^−/−^* mice with p<0.05 (one-factor ANOVA).

To investigate the role of PDK1 in AGRP neurons, we generated AGRP neuron-specific *Pdk1* knockout (*AGRPPdk1^−/−^*) mice. We found ∼90% of AGRP-positive neurons expressed PDK1 in *AGRPPdk1^+/+^* mice (Figure 1A, top panel, and 1B); in contrast, ∼90% of AGRP-positive neurons did not express PDK1 (Figure 1A, bottom panel, and 1B) in *AGRPPdk1^−/−^* mice. However, PDK1 was detected in cells that were negative for AGRP (Figure 1A, bottom panel). In a previous study, AGRP expression profiles showed transcripts in the adrenal medulla, lung, and kidney in addition to the hypothalamus [15]. We found that PDK1 expression in the adrenal gland, liver, white adipose tissue (WAT), lung, and kidney was similar in *AGRPPdk1^+/+^* and *AGRPPdk1^−/−^* mice (Methods S1 and Figure S1). These data indicated that PDK1 was inactivated specifically in AGRP neurons of *AGRPPdk1^−/−^* mice.

### Metabolic characterization of *AGRPPdk1^−/−^* mice

Both male and female *AGRPPdk1^−/−^* mice fed a normal chow diet had significantly lower body weights than control animals (Figure 1C). The naso-anal length of female *AGRPPdk1^−/−^* mice was also significantly shorter than that of control mice (Figure 1D). Computed tomography (CT) demonstrated that the calculated weight of the combined adipose tissue and visceral fat of *AGRPPdk1^−/−^* mice was significantly lower than that of control mice; however, the percent adiposity (corrected for body weight) was similar between *AGRPPdk1^−/−^* mice and control mice (Figures 1E and S2). These data demonstrated that *AGRPPdk1^−/−^* mice a smaller phenotype with a proportional reduction of fat mass. The total daily food intake of both male and female *AGRPPdk1^−/−^* mice was significantly less than that of control mice (Figure 1F). Indirect calorimetry demonstrated that the oxygen consumption and respiratory quotient (RQ) of *AGRPPdk1^−/−^* mice were similar to those of control mice (Figure 1G and 1H). The locomotor activity of *AGRPPdk1^−/−^* mice was similar to that of control mice (Figure 1I). Furthermore, the serum leptin levels of *AGRPPdk1^−/−^* mice were similar to those of control mice (data not shown). However, intraperitoneal administration of leptin to *AGRPPdk1^−/−^* mice prompted significantly greater reductions in body weight and food intake compared to control mice (Figures 1J and 1K). The serum thyroxine (data not shown), corticosterone, and norepinephrine levels of *AGRPPdk1^−/−^* mice were also similar to those of control mice (Figures S3A and S3B). These data suggested that loss of PDK1 in AGRP neurons resulted in reductions in food intake, body length and body weight and increased leptin sensitivity.

### Effects of AGRP neuron-specific PDK1 deficiency on neuropeptide expression levels

**Figure 2:**
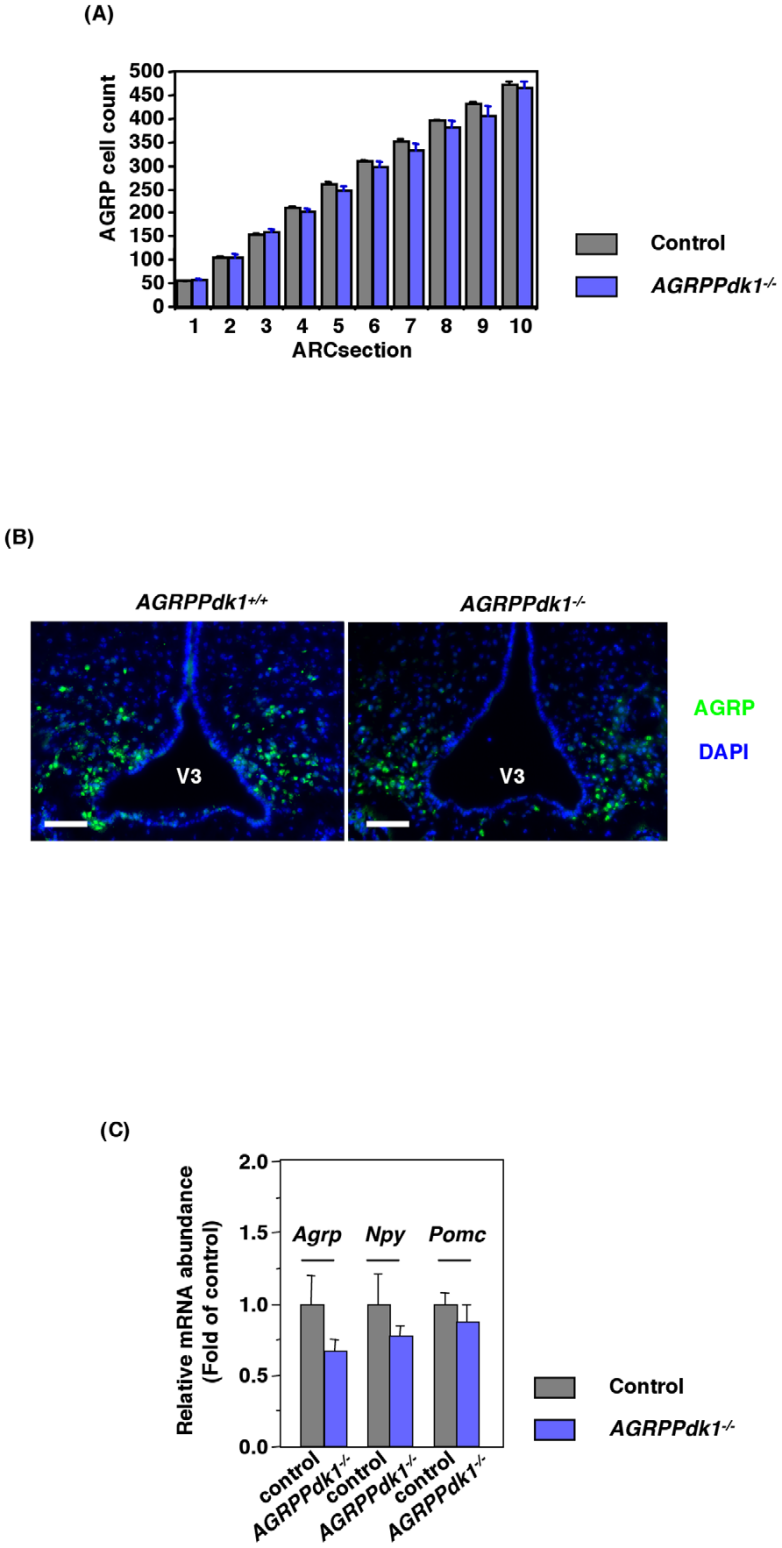
Functional defects of AGRP neurons in *AGRPPdk1^−/−^* mice. (**A**) Numbers of AGRP positive cells in the arcuate nuclei of *AGRPPdk1^+/+^* (gray bar) and *AGRPPdk1^−/−^* (blue bar) mice. AGRP cell counts indifferent regions of the arcuate nucleus showed different numbers of AGRP neurons in *AGRPPdk1^+/+^* (n = 3) and *AGRPPdk1^−/−^* mice (n = 3). (**B**) Representative Immunofluorescence images of AGRP in the hypothalamic regions of *AGRPPdk1^+/+^* (left panel) and *AGRPPdk1^−/−^* mice (right panel). Green, AGRP; blue, DAPI. Scale bars indicate 100 µm. (**C**) Expression in the fed-state of hypothalamic neuropeptide genes in control (gray bar) and *AGRPPdk1^−/−^*(blue bar) mice. Data were normalized to *β-actin* expression and represent the mean ± SEM of six mice per genotype.

We examined whether PDK1 deficiency affected the number of AGRP neurons by counting the number of anti-AGRP positive cells in serial hypothalamic sections. We found similar numbers in control and *AGRPPdk1^−/−^* mice (Figures 2A and 2B). Real-time PCR analysis of hypothalamic neuropeptide expression in control and *AGRPPdk1^−/−^* mice demonstrated similar expression levels of *Agrp*, *Npy*, and *Pomc* genes (Figure 2C). These data showed that an AGRP neuron-specific PDK1 deficiency did not affect the survival of AGRP neurons or the expression levels of *Agrp*, *Npy*, or *Pomc* in the hypothalamus.

### Melanocortin and ghrelin food intake pathways in *AGRPPdk1^−/−^* mice

**Figure 3:**
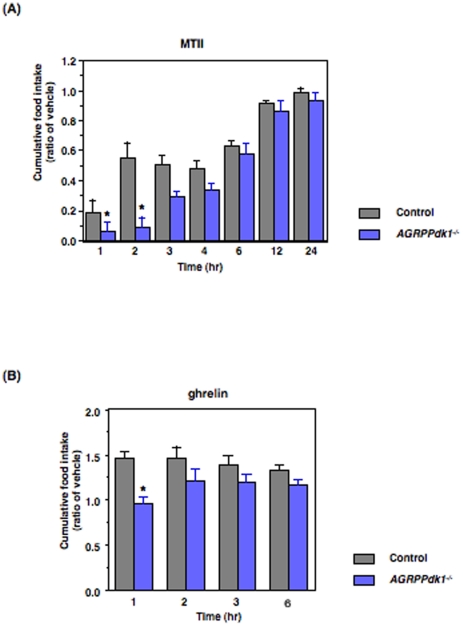
MTII and ghrelin responses in *ad libitum* fed *AGRPPdk1^−/−^*. (**A**) Cumulative food intake of male control (gray bar) and *AGRPPdk1^−/−^* (blue bar) mice injected with saline or MTII after a 24-h fast. Cumulative food intake was monitored for 24 h. Values represent the ratio of the mean cumulative food intakes of MTII: vehicle injected mice for each genotype. Asterisks indicate statistically significant differences between control and *AGRPPdk1^−/−^* mice with p<0.05 (one-factor ANOVA; n = 6 per genotype). (**B**) Cumulative food intake of male control (gray bar) and *AGRPPdk1^−/−^* (blue bar) mice injected with saline or ghrelin after a 24-h fast. Cumulative food intake was monitored for six hours. Values represent the ratio of the mean cumulative food intakes of ghrelin: vehicle injected mice for each genotype. A asterisk indicates statistically significant differences between control and *AGRPPdk1^−/−^* mice with *p<0.01 (one-factor ANOVA; n = 6 per genotype for each condition).

AGRP/NPY neurons contain AGRP, a natural antagonist of melanocortin 3 and 4 (MC3 and MC4) receptors. This antagonism reduces the anorectic effect of alpha-melanocortin stimulating hormone (α-MSH) [16] [17] [18]. To examine the melanocortin pathway in *AGRPPdk1−/−* mice, we injected mice intraperitoneally with melanotan II (MTII), a MC3/4R agonist, and measured the effect on food intake. We found that *AGRPPdk1^−/−^* mice showed greater reductions in food intake with MTII compared to control mice (Figure 3A); this indicated that the activity of the melanocortin-pathway was enhanced in *AGRPPdk1^−/−^* mice.

Administration of the growth hormone secretagogue, ghrelin, reportedly stimulates AGRP/NPY neuron activity [19] [20]. To investigate this, we delivered intraperitoneal injections of ghrelin to control and *AGRPPdk1^−/−^* mice and measured the effect on food intake. One hour after ghrelin injections, control mice showed significantly increased cumulative food intake compared to saline-injected control mice; in contrast, ghrelin-injected *AGRPPdk1^−/−^* mice demonstrated no significant changes in cumulative food intake relative to saline-injected *AGRPPdk1^−/−^* mice (Figure 3B). These data suggested that the ghrelin response system of AGRP/NPY neurons was functionally defective in *AGRPPdk1^−/−^* mice.

### Intracellular localization of Foxo1 in AGRP neurons of PDK1-deficient mice

**Figure 4:**
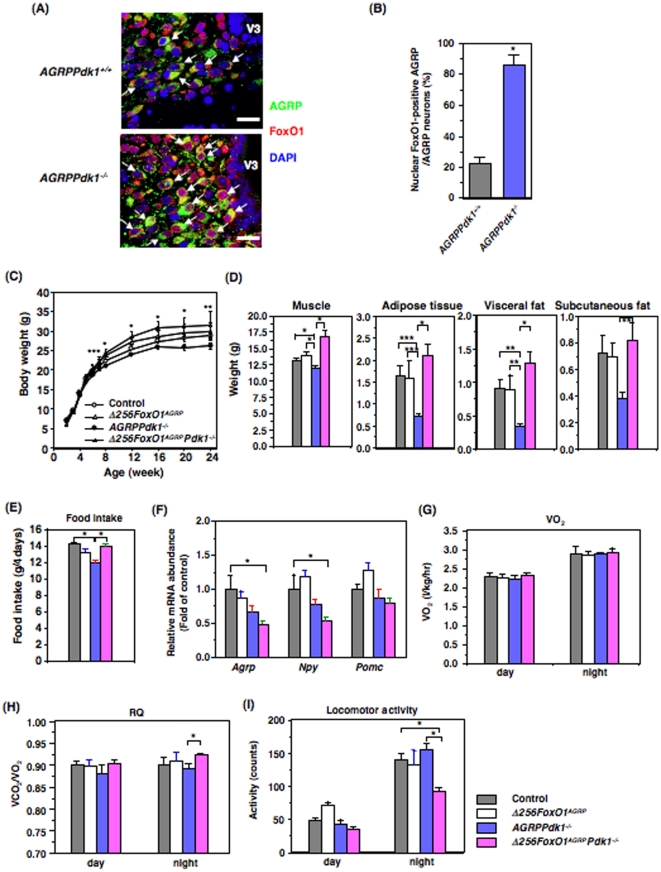
Expression of a dominant negative FoxO1 (Δ256Foxo1) rescued the metabolic phenotype of *AGRPPdk1^−/−^* mice. (**A**) Subcellular localization of Foxo1 in AGRP neurons from *AGRPPdk1^+/+^*(top panel) and *AGRPPdk1^−/−^*(bottom panel) mice. Representative double immunofluorescence images show hypothalamic regions doubly-labeled for AGRP and Foxo1. Green, AGRP; red, Foxo1; blue, DAPI staining. White arrows indicate AGRP neurons. (**B**) Quantification of nuclear Foxo1 in AGRP neurons from *AGRPPdk1^+/+^* and *AGRPPdk1^−/−^* mice. The percentage of AGRP neurons with nuclear Foxo1 among all AGRP neurons was determined as described in Experimental Procedures. Values are means ± SEM of three mice in each genotype. An asterisk indicates a statistically significant difference between *AGRPPdk1^+/+^* and *AGRPPdk1^−/−^* mice with p<0.001 (one-factor ANOVA). (**C**) Body weights of male control (open circles), *Δ256Foxo1^AGRP^* (open triangles), *AGRPPdk1^−/−^* (closed circles), and *Δ256Foxo1^AGRP^Pdk1^−/−^* (closed triangles) mice. Values represent the mean ± SEM of 20 mice in each genotype. Asterisks indicate statistically significant differences between *AGRPPdk1^−/−^* and *Δ256Foxo1^AGRP^Pdk1^−/−^* with *p<0.001, **p<0.01, and ***p<0.05 (one-factor ANOVA). (**D**) The calculated weights of muscle, adipose tissue, visceral fat, and subcutaneous fat in male control (gray bar), *Δ256Foxo1^AGRP^* (white bar), *AGRPPdk1^−/−^*(blue bar), and *Δ256Foxo1^AGRP^Pdk1* ^−/−^(magenta bar) mice at 24 weeks of age. Skeletal muscle weight, adipose tissue weight (sum of visceral and subcutaneous fats), visceral fat weight, and subcutaneous fat weight were calculated as described in Experimental Procedures. The data represent the mean weight ± SEM of 10 mice per genotype. Asterisks indicate statistically significant differences as indicated with *p<0.001, **p<0.005, and ***p<0.05 (one-factor ANOVA). (**E**) Four-day food intake for male control (gray bar), *Δ256Foxo1^AGRP^* (white bar), *AGRPPdk1^−/−^*(blue bar), and *Δ256Foxo1^AGRP^Pdk1* ^−/−^(magenta bar) mice at 24 weeks of age. The data represent the mean ± SEM of 10 mice per genotype. An asterisk indicates a statistically significant difference as indicated with *p<0.005 (one-factor ANOVA). (**F**) Expression in the fed-state of hypothalamic neuropeptide genes in control (gray bar), *Δ256Foxo1^AGRP^* (white bar), *AGRPPdk1^−/−^*(blue bar), and *Δ256Foxo1^AGRP^Pdk1* ^−/−^(magenta bar) mice. Data were normalized to *β-actin* expression and represent the mean ± SEM of six mice per genotype. An asterisk indicates a statistically significant difference between control and *Δ256Foxo1^AGRP^Pdk1* ^−/−^ mice with p<0.05 (one-factor ANOVA). (**G**) Oxygen consumption in male control (gray bar), *Δ256Foxo1^AGRP^* (white bar), *AGRPPdk1^−/−^*(blue bar), and *Δ256Foxo1^AGRP^Pdk1* ^−/−^(magenta bar) mice at 24 weeks of age. The values represent the mean ± SEM of 10 male mice per genotype. (**H**) Respiratory quotient of male control (gray bar), *Δ256Foxo1^AGRP^* (white bar), *AGRPPdk1^−/−^*(blue bar), and *Δ256Foxo1^AGRP^Pdk1* ^−/−^(magenta bar) mice at 24 weeks of age. The values represent the mean ± SEM of 10 mice per genotype. An asterisk indicates a statistically significant difference between *AGRPPdk1^−/−^* and *Δ256Foxo1^AGRP^Pdk1* ^−/−^ mice with p<0.05 (one-factor ANOVA). (**I**) Basal locomotor activity for male control (gray bar), *Δ256Foxo1^AGRP^* (white bar), *AGRPPdk1^−/−^*(blue bar), and *Δ256Foxo1^AGRP^Pdk1* ^−/−^(magenta bar) mice at 24 weeks of age during the light and dark phases. The values represent the mean ± SEM of 5 mice per genotype. An asterisk indicates a statistically significant difference as indicated with p<0.001 (one-factor ANOVA).

The Foxo1 protein is phosphorylated in a PI3-kinase/PDK1/Akt-dependent manner [21]. Furthermore, in AGRP neurons, Foxo1 is regulated by feeding status [22], [23]. Therefore, we investigated whether PDK1-deficient AGRP neurons exhibited aberrant Foxo1 localization. We found that the endogenous Foxo1 was localized mainly in the cytosol of AGRP neurons in *AGRPPdk1^+/+^* mice (Figure 4A, top panel). In contrast, Foxo1 was localized to the nucleus in approximately 80% of AGRP neurons in *AGRPPdk1^−/−^* mice (Figure 4A, bottom panel, and 4B).

### Transactivation-defective Foxo1 (Δ256Foxo1) reverses the *AGRPPdk1^−/−^* phenotype

To investigate whether Foxo1 acts downstream of PDK1 in AGRP neurons, we generated transgenic mice that expressed transactivation-defective Δ256Foxo1 [7] specifically in AGRP neurons (*Δ256Foxo1^AGRP^*mice). From the above study, Foxo1 in AGRP neurons of *AGRPPdk1^−/−^* mice is localized mostly in nucleus and might be activated. Therefore, it is speculated that expression of Δ256Foxo1 inhibits endogenous Foxo1 in AGRP neurons and rescues phenotypes of *AGRPPdk1^−/−^* mice. These animals were obtained at the expected Mendelian ratio (approximately 25%). To confirm that the FLAG-Δ256Foxo1 protein was expressed in the AGRP neurons of *Δ256Foxo1^AGRP^*mice, we performed double-immunofluorescence with anti-FLAG and anti-AGRP antibodies. The results demonstrated that Δ256Foxo1 was expressed in AGRP neurons (Figure S4A). Approximately 80% of the cells that stained for AGRP also stained for FLAG in *Δ256Foxo1^AGRP^*mice (Figure S4B). FLAG-Δ256Foxo1 was localized to the nucleus (Figure S4A). FLAG-Δ256Foxo1 expression was not detected in Western blots of total hypothalamic lysates from *Δ256Foxo1^AGRP^*mice due to the restricted expression of FLAG-Δ256Foxo1 in defined subpopulations of the hypothalamic neurons (Figure S4C). Furthermore, no expression of FLAG-Δ256Foxo1 was detected in peripheral tissues, including WAT, liver, lung, kidney, and adrenal gland (Figure S4C). These data indicated that FLAG-Δ256Foxo1 was expressed specifically in AGRP neurons. The *Δ256Foxo1^AGRP^*mice exhibited no hypothalamic phenotype; they had normal body weight, adiposity, food intake, oxygen consumption, respiratory quotient, and locomotor activity (Figure 4C–4H).

To examine whether Foxo1 acted downstream of PDK1 in AGRP neurons, we crossed *Δ256Foxo1^AGRP^*mice with *Pdk1^flox/+^* mice to generate *Δ256Foxo1^AGRP^* - *AGRPPdk1^+/−^* (*Δ256Foxo1^AGRP^Pdk1^+/−^*) mice. These mice were crossed with *Pdk1^flox/+^* to generate *Δ256Foxo1^AGRP^Pdk1^−/−^* mice (Figure S5). Surprisingly, *Δ256Foxo1^AGRP^Pdk1^−/−^* mice had significantly greater body weight than control and *AGRPPdk1^−/−^* mice (Figure 4C). CT scans demonstrated that *Δ256Foxo1^AGRP^Pdk1^−/−^* mice had significantly greater lean body mass and visceral fat mass than control and *AGRPPdk1^−/−^* mice (Figure 4D and S2). The food intake of *Δ256Foxo1^AGRP^Pdk1^−/−^* mice was significantly greater than that of *AGRPPdk1^−/−^* mice (Figure 4E). In the fed-state, hypothalamic *Agrp* and *Npy* expression levels in *Δ256Foxo1^AGRP^Pdk1^−/−^* mice were lower than those of *AGRPPdk1^−/−^* mice (Figure 4F). Indirect calorimetry showed that, although oxygen consumption was similar for *Δ256Foxo1^AGRP^Pdk1^−/−^* and control mice, the RQ of *Δ256Foxo1^AGRP^Pdk1^−/−^* mice was significantly greater than that of control mice (Figures 4G and 4H). In addition, 24-week-old *Δ256Foxo1^AGRP^Pdk1^−/−^* mice exhibited significantly less locomotor activity than control mice, particularly during the dark phase (Figures 4I). The glucose tolerance and insulin sensitivity of *Δ256Foxo1^AGRP^Pdk1^−/−^* mice were similar to those of *AGRPPdk1^−/−^* mice (Figure S6A and S6B). These data suggested that overexpression of a transactivation-defective *Δ*256Foxo1 in AGRP neurons reversed the hypothalamic phenotypes of *AGRPPdk1^−/−^* mice.

### Energy expenditure in calorie-restricted *AGRPPdk1^−/−^* and *Δ256Foxo1^AGRP^Pdk1^−/−^* mice

**Figure 5:**
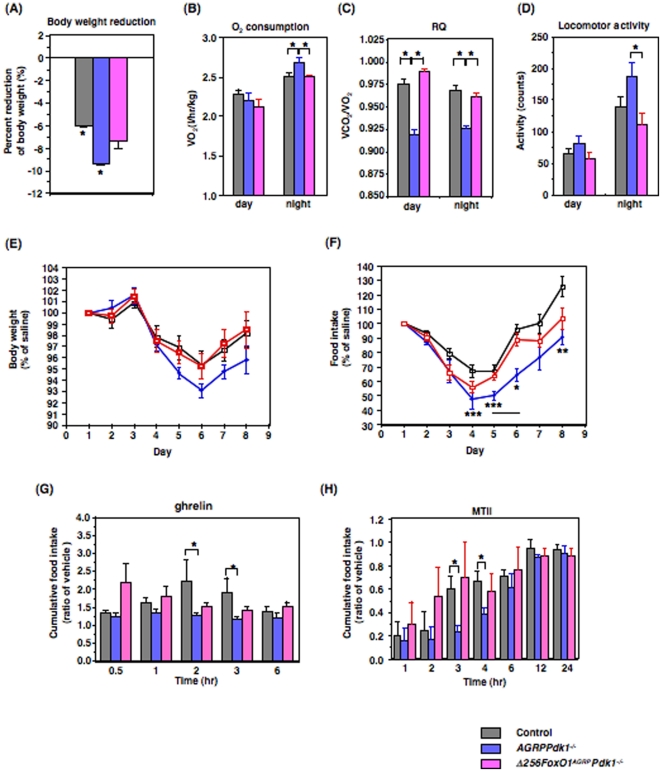
Metabolic phenotypes of calorie-restricted *AGRPPdk1^−/−^* and *Δ256Foxo1^AGRP^Pdk1* ^−/−^ mice. (**A**) Effects of 4 weeks of calorie restriction on body weight of 20-week-old male control (gray bar), *AGRPPdk1^−/−^*(blue bar), and *Δ256Foxo1^AGRP^Pdk1* ^−/−^(magenta bar) mice. Values represent means ± SEM of the percentages of reduction of body weight at the end (4 week) of pair feeding compared to at the beginning of pair feeding. An asterisk indicates a statistically significant difference between bodyweight at the beginning and at the end of calorie restriction with p<0.05 (one-factor ANOVA; n = 10 male mice per genotype). (**B**) Oxygen consumption of male calorie-restricted control (gray bar), *AGRPPdk1^−/−^* (blue bar), and *Δ256Foxo1^AGRP^Pdk1* ^−/−^(magenta bar) during the light and dark phases. Values represent the mean ± SEM of 10 mice per genotype. An asterisk indicates a statistically significant difference between *AGRPPdk1^−/−^* and control or *Δ256Foxo1^AGRP^Pdk1* ^−/−^ mice with p<0.05 (one-factor ANOVA). (**C**) Respiratory quotients of male pair-fed control (gray bar), *AGRPPdk1^−/−^* (blue bar), and *Δ256Foxo1^AGRP^Pdk1* ^−/−^ (magenta bar) mice during the light and dark phases. Values represent the mean ± SEM of 10 mice per genotype. An asterisk indicates a statistically significant difference between *AGRPPdk1^−/−^* and control or *Δ256Foxo1^AGRP^Pdk1* ^−/−^ mice with p<0.001 (one-factor ANOVA). (**D**) Basal locomotor activity of male pair-fed control (gray bar), *AGRPPdk1^−/−^* (blue bar), and *Δ256Foxo1^AGRP^Pdk1* ^−/−^ (magenta bar) during the light and dark phases. Values represent the mean ± SEM of 10 mice per genotype. An asterisk indicates a statistically significant difference between *AGRPPdk1^−/−^* and *Δ256Foxo1^AGRP^Pdk1* ^−/−^ mice with p<0.05 (one-factor ANOVA). (**E–F**) Effects of intraperitoneal leptin injections on body weight and food intake of pair-fed control (open squares, n = 10), *AGRPPdk1^−/−^* mice (blue closed circles, n = 10), and *Δ256Foxo1^AGRP^Pdk1* ^−/−^(red squares, n = 10) mice. Leptin was injected on 3 consecutive days. Body weight (E) and food intake (F) were monitored for eight days. Body weight and food intake data are presented as the percentage of that for saline-injected mice for each genotype. An asterisk in (F) indicates statistically significant differences between control and *AGRPPdk1^−/−^* mice with *p<0.001, **p<0.01, and ***p<0.05 by one-factor ANOVA. (**G**) Cumulative food intake for male pair-fed control (gray bar), *AGRPPdk1^−/−^* (blue bar), and *Δ256Foxo1^AGRP^Pdk1* ^−/−^ (magenta bar) mice injected with saline or ghrelin after a 24-h fast. Cumulative food intake was monitored for 6 h. Values represent the ratio of the mean cumulative food intakes of ghrelin:vehicle injected mice for each genotype. An asterisk indicates statistically significant differences between control and *AGRPPdk1^−/−^* mice with *p<0.02 (one-factor ANOVA; n = 10 per genotype for each condition). (**H**) Cumulative food intake of male pair-fed control (gray bar), *AGRPPdk1^−/−^* (blue bar), and *Δ256Foxo1^AGRP^Pdk1* ^−/−^ (magenta bar) mice injected with saline or MTII after a 24-h fast. Cumulative food intake was monitored for 24 h. Values represent the ratio of the mean cumulative food intakes of MTII:vehicle injected mice for each genotype. Asterisks indicate statistically significant differences between control and *AGRPPdk1^−/−^* mice with p<0.05 (one-factor ANOVA; n = 10 per genotype).

To explore the relative contributions of food intake and locomotor activity to the increased body weight of *Δ256Foxo1^AGRP^Pdk1^−/−^* mice, we performed a calorie-restricted study with 20-week-old mice fed a normal chow diet. After 4 weeks of calorie-restricted feeding, control and *AGRPPdk1^−/−^* mice showed significantly reduced body weights compared to *Δ256Foxo1^AGRP^Pdk1^−/−^* mice (Figure 5A). The body weight of *Δ256Foxo1^AGRP^Pdk1^−/−^* mice also decreased compared to that observed on an *ad libitum* diet, but it was not statistically significantly (Figure 5A). Indirect calorimetry demonstrated that, during the dark phase, the oxygen consumption of *AGRPPdk1^−/−^* mice was significantly greater than control and *Δ256Foxo1^AGRP^Pdk1^−/−^* mice (Figure 5B); moreover, the respiratory quotient of *AGRPPdk1^−/−^* mice was significantly lower than control and *Δ256Foxo1^AGRP^Pdk1^−/−^* mice during both the light and dark phases (Figure 5C). In addition, *AGRPPdk1^−/−^* mice exhibited significantly greater locomotor activity than control and *Δ256Foxo1^AGRP^Pdk1^−/−^* mice, particularly during the dark phase (Figure 5D). These data indicated that the increased body weight of *Δ256Foxo1^AGRP^Pdk1^−/−^* mice, compared to *AGRPPdk1^−/−^* mice, resulted from increased food intake, reduced oxygen consumption, and reduced locomotive activity.

We also examined the responses to leptin, ghrelin, and MTII under pair-feeding conditions to confirm the functional defects of AGRP neurons in *AGRPPdk1^−/−^* mice and the functional normalization of AGRP neurons in *Δ256Foxo1^AGRP^Pdk1^−/−^* mice. Intraperitoneal administration of leptin prompted significantly greater reductions in body weight and food intake in *AGRPPdk1^−/−^* mice compared to control and *Δ256Foxo1^AGRP^Pdk1^−/−^* mice; although the changes in body weight of *AGRPPdk1^−/−^* mice were not significant (Figures 5E and 5F). These data confirmed the finding under normal feeding conditions that *AGRPPdk1^−/−^* mice had increased leptin sensitivity. Intraperitoneal injections of ghrelin prompted significantly increased cumulative food intake in controls compared to *AGRPPdk1^−/−^* mice, similar to the findings under normal feeding conditions. Furthermore, ghrelin-injected *Δ256Foxo1^AGRP^Pdk1^−/−^* mice demonstrated no significant differences in cumulative food intake compared to control mice (Figure 5G). Finally, intraperitoneal MTII injections showed that *AGRPPdk1^−/−^* mice were more sensitive to MTII compared to control mice; the effects were similar to those observed under normal feeding conditions. In contrast, *Δ256Foxo1^AGRP^Pdk1^−/−^* mice showed MTII responses similar to control mice (Figure 5H). These data suggested that the ghrelin response in AGRP neurons of *AGRPPdk1^−/−^* mice was functionally defective and the activity of the melanocortin-pathway was enhanced in *AGRPPdk1^−/−^* mice under pair-fed conditions.

### Leptin inhibition of ghrelin-induced Ca^2+^ influx in PDK1-deficient AGRP neurons

**Figure 6:**
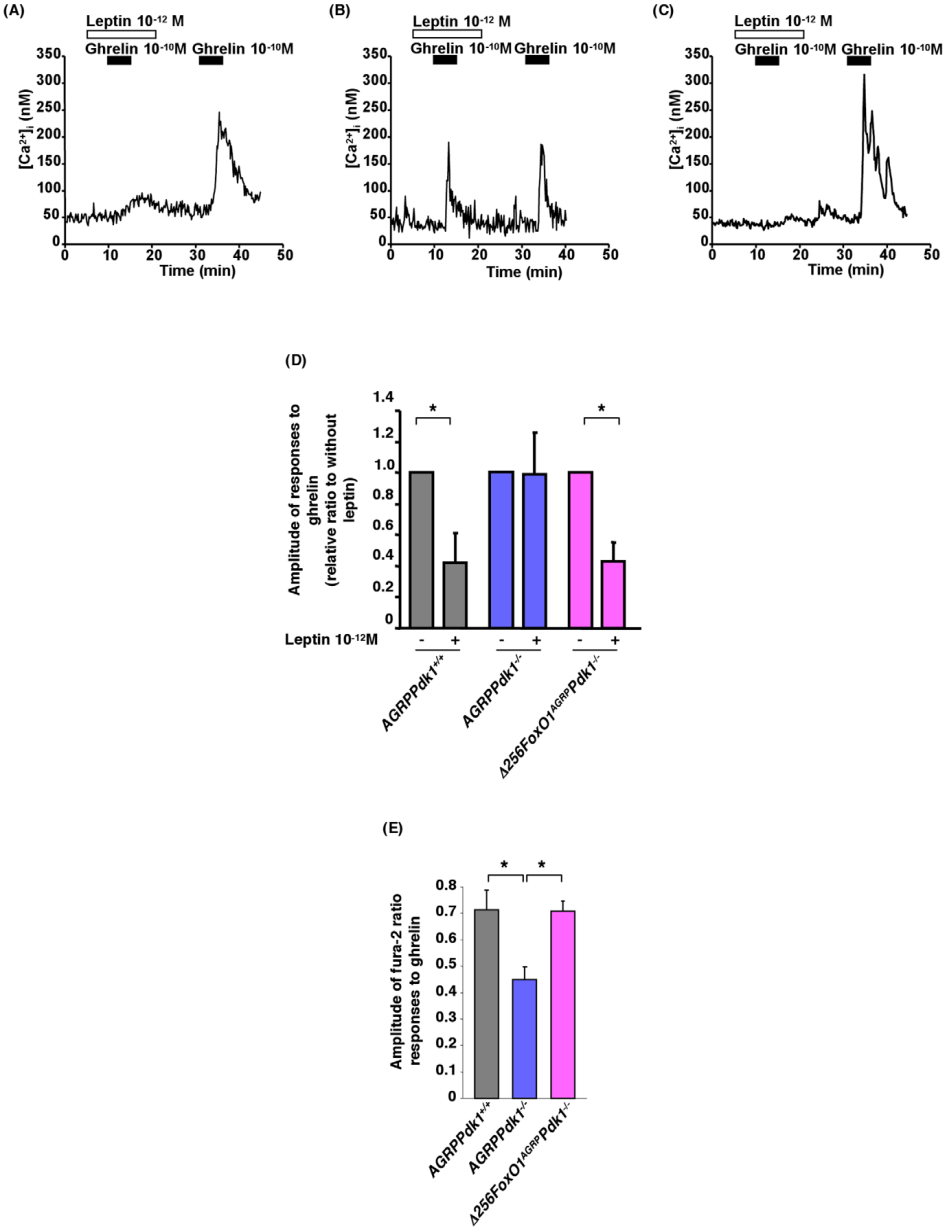
Functional defects of AGRP neurons in *AGRPPdk1^−/−^* mice. (**A**)–(**C**) Administration of 10^−10^ M of ghrelin increased cytosolic calcium ([Ca^2+^]_i_) in AGRP neurons from *AGRPPdk1^+/+^* (A), *AGRPPdk1^−/−^* (B), and *Δ256Foxo1^AGRP^Pdk1* ^−/−^ (C) mice. Administration of 10^−12^ M of leptin inhibited increases in [Ca^2+^]_i_ induced by ghrelin in *AGRPPdk1^+/+^* (A) and *Δ256Foxo1^AGRP^Pdk1* ^−/−^ (B) neurons, but not in *AGRPPdk1^−/−^* neurons (C). (**D**) Amplitude of [Ca^2+^]_i_ responses to ghrelin in the absence or presence of leptin (10^−12^ M) in AGRP neurons of *AGRPPdk1^+/+^*(gray bar), *AGRPPdk1^−/−^*(blue bar), and *Δ256Foxo1^AGRP^Pdk1* ^−/−^(magenta bar) mice. Values are means ± SEM of the ratio of [Ca^2+^]_i_ in the presence and absence of leptin. An asterisk indicates a statistically significant difference between the absence and the presence of leptin with p<0.05 (one-factor ANOVA: n = 10 AGRP neurons per genotype). (**E**) Fura-2 detection of [Ca^2+^]_i_ in response to ghrelin. Values are means ± SEM of ghrelin-stimulated fura-2 ratios in AGRP neurons from *AGRPPdk1^+/+^*(gray bar), *AGRPPdk1^−/−^*(blue bar), and *Δ256Foxo1^AGRP^Pdk1* ^−/−^(magenta bar). An asterisk indicates a statistically significant difference between *AGRPPdk1^−/−^* and control or *Δ256Foxo1^AGRP^Pdk1* ^−/−^ mice with p<0.05 (one-factor ANOVA: n = 10 AGRP neurons per genotype).

It has been demonstrated that ghrelin (10^−12^ to 10^−8^ mol/l) increased cytosolic Ca^2+^ [Ca^2+^]_i_ in most NPY-containing neurons and that leptin inhibited ghrelin-induced changes in [Ca^2+^]_i_ [24]. Often, depolarization of the plasma membrane triggers an increase in [Ca^2+^]_i_ that signals the release of neurotransmitters or hormones and regulation of gene expression [25] [26]. Therefore, increases in [Ca^2+^]_i_ are good indicators of neuronal activity. To investigate the effect of PDK1 deficiency in AGRP neurons, we measured ghrelin-induced changes in [Ca^2+^]_i_ with fura-2 fluorescence imaging. Single neurons isolated from *AGRPPdk1^+/+^* mice exhibited increased [Ca^2+^]_i_ in response to ghrelin (10^−10^ M); furthermore, leptin (10^−12^ M) inhibited ghrelin-induced changes in [Ca^2+^]_i_ (Figures 6A and 6D). In contrast, single neurons isolated from *AGRPPdk1^−/−^* mice exhibited significantly attenuated changes in [Ca^2+^]_i_ in response to ghrelin (Figures 6B and 6E); moreover, leptin did not inhibit ghrelin-induced changes in [Ca^2+^]_i_ (Figures 6B and 6D). These data suggested that PDK1 mediated both ghrelin and leptin activities in AGRP neurons.

Interestingly, in single AGRP neurons from *Δ256Foxo1^AGRP^Pdk1^−/−^* mice, leptin inhibited ghrelin-induced increases in [Ca^2+^]_i_ to the extent observed in control mice (Figures 6C and 6D). Furthermore, the amplitude of the [Ca^2+^]_i_ response to ghrelin was restored to the level observed in control mice (Figure 6E). These data suggested that overexpression of Δ256Foxo1 restored the [Ca^2+^]_i_ response to ghrelin in AGRP neurons of *AGRPPdk1^−/−^* mice.

## Discussion

PDK1 regulates cell survival, cell cycle control, protein translation, and glucose metabolism [27] [28] [29] [30]. Disruption of PDK1 caused apoptosis in pancreatic β-cells, pituitary corticotrophs, or cardiomyocytes, but not in hepatocytes or POMC neurons [31] [32] [5] [33]. That is, PDK1 regulates cell survival in a tissue-specific and time-dependent manner. In this study, disruption of PDK1 in AGRP neurons by the Cre recombinase gene, under the control of the *Agrp* promoter, occurred at 2–3 weeks of age [10] and did not cause apoptosis of AGRP neurons. Therefore, phenotypes of *AGRPPdk1^−/−^* mice were derived from functional defects, but not loss of AGRP neurons.

Our data indicated that PDK1 was indispensable for the orexigenic activity of AGRP neurons. Hypothalamic AGRP neurons express AGRP, NPY, the neurotransmitter GABA [34], and potentially other undiscovered molecules. In *AGRPPdk1^−/−^* mice, the expression of *Agrp* and *Npy* tended to be lower than that observed in control mice although the difference was not significant. Interestingly, the *Δ256Foxo1^AGRP^Pdk1^−/−^* mice exhibited significantly increased food intake compared to *AGRPPdk1^−/−^* mice in spite of significantly decreased expression of *Agrp* and *Npy*. Therefore, changes in the expression levels of *Agrp* and *Npy* may not explain the changes in food intake in *AGRPPdk1^−/−^* mice. This conclusion is consistent with previous reports that *Agrp* and *Npy* knockout mice showed no apparent phenotypes for food intake or body weight regulation [35] [36]. In contrast, defects in AGRP neurons in adult mice, but not in neonatal mice, caused rapid starvation. This finding suggested that, for feeding and weight gain, AGRP neurons were more important than the neuropeptides they released (AGRP or NPY) [37] [38] [39]. Recently, it has been reported that mice in which p110β was inactivated in AGRP neurons were found to display an age-dependent lean phenotype with reduced adiposity, hypoleptinemia, and resistance to diet-induced obesity, phenotypes similar to *AGRPPdk1^−/−^* mice [40]. These findings suggest an important role of PI(3)K-PDK1 in AGRP neurons for long-term regulation of energy homeostasis.

A recent study showed that the AGRP neuron-specific knockout of the vesicular GABA transporter (*Vgat*) caused reduced synaptic release of GABA; these knockout mice were lean, resistant to obesity, had increased locomotor activity, and had an attenuated hyperphagic response to ghrelin [41]. These phenotypes were similar to *AGRPPdk1^−/−^* mice, suggesting that, in AGRP neurons, a PDK1-deficiency may be involved in reducing the release of GABA. Indeed, an increase in cytosolic [Ca2+]i triggers the release of GABA from neurons [42]. Our results showed that single neurons isolated from *AGRPPdk1^−/−^* mice exhibited significantly attenuated changes in [Ca^2+^]_i_ responses to ghrelin; furthermore, the ghrelin-induced change in [Ca^2+^]_i_ was not inhibited by leptin. Interestingly, the above aberrant [Ca^2+^]_i_ responses to ghrelin and leptin in AGRP neurons of *AGRPPdk1^−/−^* mice were restored by expression of Δ256Foxo1 in AGRP neurons. The mutant Δ256Foxo1 had a dominant negative effect on some of the Foxo1-target genes, suggesting that transcriptional regulations by Foxo1 were important for the ghrelin-induced [Ca^2+^]_i_ responses in AGRP neurons.

We did not observe a phenotype specific to *Δ256Foxo1^AGRP^* mice. Δ256Foxo1 still has two intact Akt phosphorylation sites, Threonine 24 and Serine 253 [7]. Phosphorylation of the first and second Akt phosphorylation sites can affect DNA binding and regulate transcriptional activity of Foxos [43] [44] [45] [46]. Therefore, loss-of-PDK1 may enhance DNA binding of Δ256Foxo1. In contrast, in the presence of intact PDK1, Δ256Foxo1 may be phosphorylated in the nucleus and unable to bind to DNA, leading to no specific phenotype for *Δ256Foxo1^AGRP^* mice.

AGRP neurons send dense projections to other neurons including POMC neurons [47] [48]. The release of GABA from AGRP neurons has an inhibitory effect on POMC neurons [41], [49]; thus, a PDK1-deficiency in AGRP neurons may lead to disinhibition of POMC neurons. Indeed, in the present study, *AGRPPdk1^−/−^* mice exhibited increased sensitivity to MTII, an MC3/4R agonist, and an attenuated ghrelin-induced increase in food intake. These data suggested that output from AGRP neurons led to tonic inhibition of the melanocortin receptor.

We observed that the ghrelin response system of AGRP/NPY neurons was functionally defective in *AGRPPdk1^−/−^* mice and single neurons isolated from *AGRPPdk1^−/−^* mice exhibited significantly attenuated changes in [Ca^2+^]_I_ in response to ghrelin. However, intracerebroventricular injection of ghrelin in control mice didn't induced phosphorylation of PDK1 (Figure S7). The growth hormone secretagogue receptor type 1 a (GHS-R1a) transduces the main signal carried by ghrelin [50] and the binding of ghrelin to GHS-R1a has been shown to trigger Akt phosphorylation [51]. Furthermore, it was recently demonstrated that early G_i/o_-proteins-dependent PI(3)K activation leads to membrane recruitment of Akt, which phosphorylated c-Src with subsequent phosphorylation at T308 and S473 by PDK1 and mTORC2, respectively [52]. Therefore, although ghrelin doesn't phosphorylate PDK1 directly, PDK1 is located downstream of ghrelin signaling.

In this study, we demonstrated that PDK1 and Foxo1 played important roles in regulating energy homeostasis that were independent of the expression of several known neuropeptides in AGRP neurons. Further studies are needed to identify the mechanism underlying Foxo1 regulation of energy expenditure.

## Supporting Information

Figure S1 **Expression of PDK1 in hypothalamus and peripheral tissues.** Western blot analysis was performed as described in Experimental Procedures. PDK1 and tubulin (loading control) levels are shown for the adrenal gland, liver, white adipose tissue (WAT), lung, and kidney of *AGRPPdk1^+/+^* (lane 1 and 2) and *AGRPPdk1^−/−^* (lane 3 and 4) mice.
(TIF)

Figure S2 **Body composition of** ***AGRPPdk1^−/−^*** **and** ***Δ256Foxo1^AGRP^Pdk1*** **^−/−^.** Adiposity of male control (gray bar), *Δ256FoxO1^AGRP^* (white bar), *AGRPPdk1^−/−^*(blue bar), and *Δ256Foxo1^AGRP^Pdk1* ^−/−^(magenta bar) mice at 24 weeks of age. Skeletal muscle mass, adipose tissue mass (sum of visceral and subcutaneous fats), visceral fat mass, and subcutaneous fat mass were calculated as described in Experimental Procedures. The data represent the mean percent body weight ± SEM of 10 mice per genotype. Asterisks indicate statistically significant differences between *Δ256Foxo1^AGRP^Pdk1* ^−/−^ and control, *Δ256FoxO1^AGRP^*, or *AGRPPdk1^−/−^* with *p<0.001 and **p<0.05 (one-factor ANOVA).
(TIF)

Figure S3 **Serum corticosterone and norepinephrine levels.** (**A**) Serum corticosterone levels of control (gray bar), *Δ256Foxo1* ***^AGRP^*** (white bar), *AGRPPdk1^−/−^*(blue bar), and *Δ256Foxo1^AGRP^Pdk1* ^−/−^(magenta bar) mice in fed and fasted states. (**B**) Serum norepinephrine levels of control (gray bar), *Δ256Foxo1^AGRP^* (white bar), *AGRPPdk1^−/−^*(blue bar), and *Δ256Foxo1^AGRP^Pdk1* ^−/−^(magenta bar) mice.
(TIF)

Figure S4 **Expression of FLAG-Δ256Foxo1 in** ***Δ256Foxo1^AGRP^*** **mice.** (**A**) Representative double immunofluorescence images of AGRP and FLAG in the arcuate nucleus of 12-week-old *Δ256Foxo1^AGRP^* mice fed *ad libitum*. Green, AGRP: red, FLAG: blue, DAPI. White arrows indicate AGRP neurons in which FLAG-Δ256Foxo1 is expressed. Scale bars indicate 50 µm. (**B**) Quantification of FLAG-Δ256Foxo1 expression in AGRP neurons. FLAG staining was assessed in at least 50 AGRP-positive neurons from three mice. Results are expressed as the means (± SEM) percentage of AGRP-positive cells in which FLAG-Δ256Foxo1 expression was detected. (**C**) Western blot analysis of FLAG and tubulin (loading control) in the hypothalamus (HYP), white adipose tissue (WAT), lung (LU), liver (LIV), adrenal gland (AG), and kidney (KID) of *Δ256Foxo1^AGRP^*. The WAT of *Δ256Foxo1^aP2^* served as a positive control (Methods S1).
(TIF)

Figure S5 **Breeding strategy for targeted deletion of** ***Pdk1*** **and expression of Δ256Foxo1 in AGRP neurons.**
(TIF)

Figure S6 **Glucose metabolism in** ***AGRPPdk1^−/−^*** **and** ***Δ256Foxo1^AGRP^Pdk1*** **^−/−^ mice.** (**A**) Mean (±SEM) glucose tolerance in *AGRPPdk1^−/−^* (open circles) and *Δ256Foxo1^AGRP^Pdk1^−/−^* (closed circles) mice at 24 weeks of age: n = 10 mice per genotype. (**B**) Mean (±SEM) insulin tolerance in *AGRPPdk1^−/−^* (open circles) and *Δ256Foxo1^AGRP^Pdk1^−/−^* (closed circles) mice at 24 weeks of age: n = 10 mice per genotype.
(TIF)

Figure S7 **Ghrelin doesn't phosphorylate PDK1 in AGRP neurons.** Representative immunofluorescence images of phospho-PDK1 in the hypothalamus of 13-week-old C57bl6 mice after a 24-hour fast (top panel), after an intracerebroventricular injection of ghrelin (bottom panel) (Methods S1). Green, red and blue indicate AGRP, phospho-PDK1, and DAPI staining, respectively.
(TIF)

Methods S1 (DOC)
