# Supplementary figures and images for: PDK1-Foxo1 in Agouti-Related Peptide Neurons Regulates Energy Homeostasis by Modulating Food Intake and Energy Expenditure
Source: PLoS One. 2011 Apr 7;6(4):e18324. doi: 10.1371/journal.pone.0018324 (PMC3072380; doi:10.1371/journal.pone.0018324)

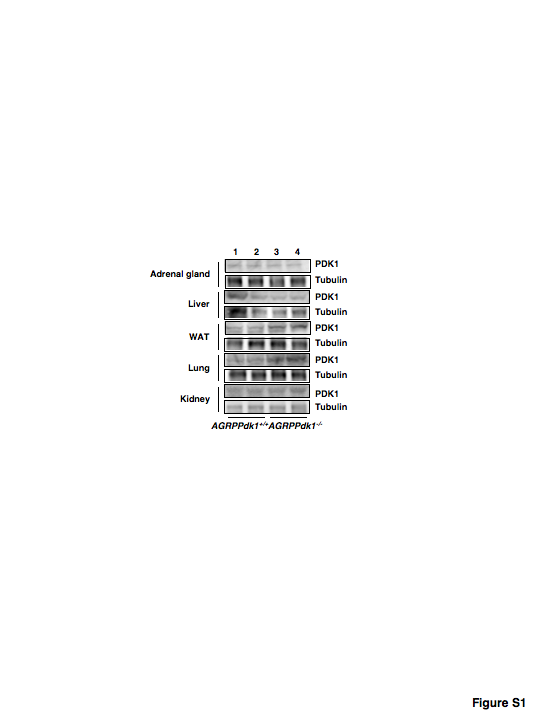

Supplement: Figure S1 — Expression of PDK1 in hypothalamus and peripheral tissues. Western blot analysis was performed as described in Experimental Procedures. PDK1 and tubulin (loading control) levels are shown for the adrenal gland, liver, white adipose tissue (WAT), lung, and kidney of AGRPPdk1+/+ (lane 1 and 2) and AGRPPdk1−/− (lane 3 and 4) mice. (TIF) [file pone.0018324.s001.tif]

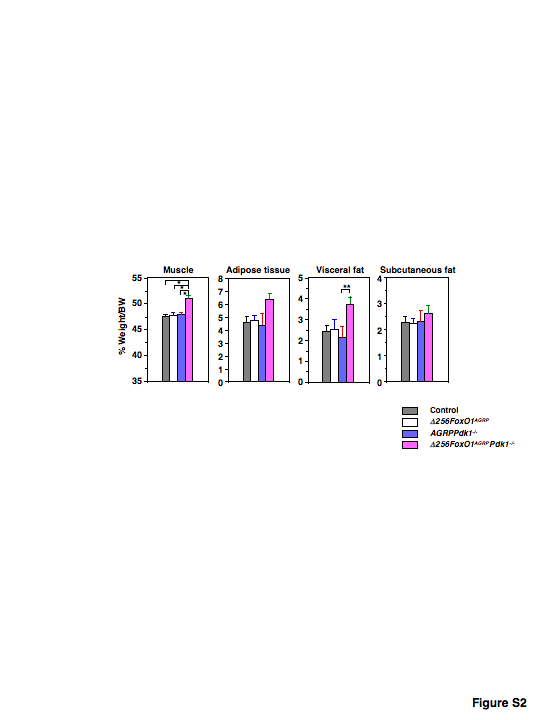

Supplement: Figure S2 — Body composition of AGRPPdk1−/− and Δ256Foxo1AGRPPdk1 −/−. Adiposity of male control (gray bar), Δ256FoxO1AGRP (white bar), AGRPPdk1−/−(blue bar), and Δ256Foxo1AGRPPdk1 −/−(magenta bar) mice at 24 weeks of age. Skeletal muscle mass, adipose tissue mass (sum of visceral and subcutaneous fats), visceral fat mass, and subcutaneous fat mass were calculated as described in Experimental Procedures. The data represent the mean percent body weight ± SEM of 10 mice per genotype. Asterisks indicate statistically significant differences between Δ256Foxo1AGRPPdk1 −/− and control, Δ256FoxO1AGRP, or AGRPPdk1−/− with *p<0.001 and **p<0.05 (one-factor ANOVA). (TIF) [file pone.0018324.s002.tif]

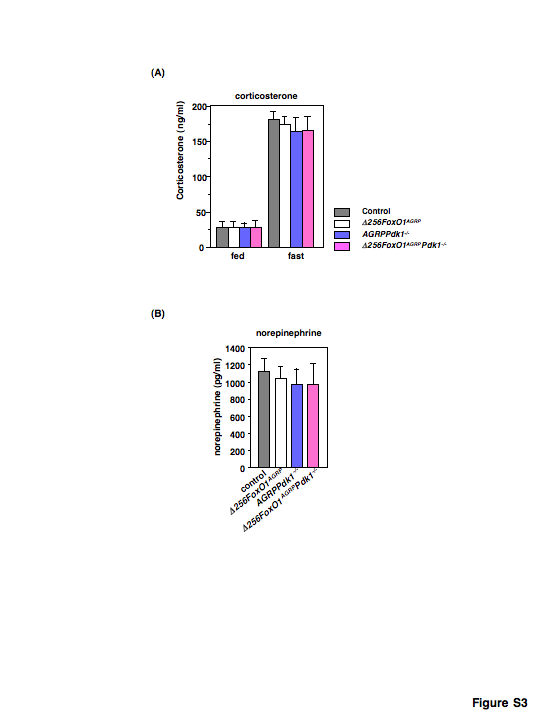

Supplement: Figure S3 — Serum corticosterone and norepinephrine levels. (A) Serum corticosterone levels of control (gray bar), Δ256Foxo1 AGRP (white bar), AGRPPdk1−/−(blue bar), and Δ256Foxo1AGRPPdk1 −/−(magenta bar) mice in fed and fasted states. (B) Serum norepinephrine levels of control (gray bar), Δ256Foxo1AGRP (white bar), AGRPPdk1−/−(blue bar), and Δ256Foxo1AGRPPdk1 −/−(magenta bar) mice. (TIF) [file pone.0018324.s003.tif]

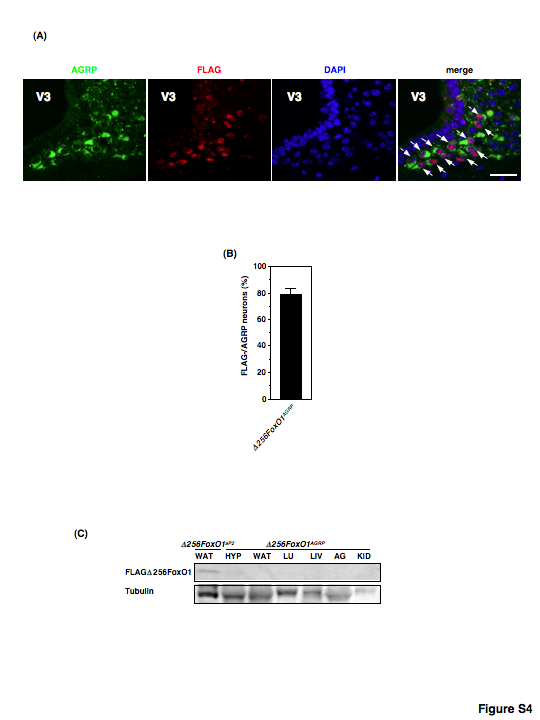

Supplement: Figure S4 — Expression of FLAG-Δ256Foxo1 in Δ256Foxo1AGRP mice. (A) Representative double immunofluorescence images of AGRP and FLAG in the arcuate nucleus of 12-week-old Δ256Foxo1AGRP mice fed ad libitum. Green, AGRP: red, FLAG: blue, DAPI. White arrows indicate AGRP neurons in which FLAG-Δ256Foxo1 is expressed. Scale bars indicate 50 µm. (B) Quantification of FLAG-Δ256Foxo1 expression in AGRP neurons. FLAG staining was assessed in at least 50 AGRP-positive neurons from three mice. Results are expressed as the means (± SEM) percentage of AGRP-positive cells in which FLAG-Δ256Foxo1 expression was detected. (C) Western blot analysis of FLAG and tubulin (loading control) in the hypothalamus (HYP), white adipose tissue (WAT), lung (LU), liver (LIV), adrenal gland (AG), and kidney (KID) of Δ256Foxo1AGRP. The WAT of Δ256Foxo1aP2 served as a positive control (Methods S1). (TIF) [file pone.0018324.s004.tif]

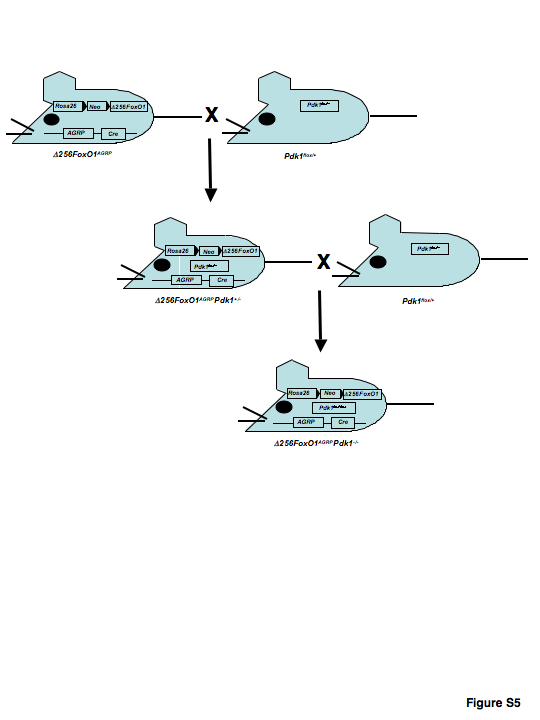

Supplement: Figure S5 — Breeding strategy for targeted deletion of Pdk1 and expression of Δ256Foxo1 in AGRP neurons. (TIF) [file pone.0018324.s005.tif]

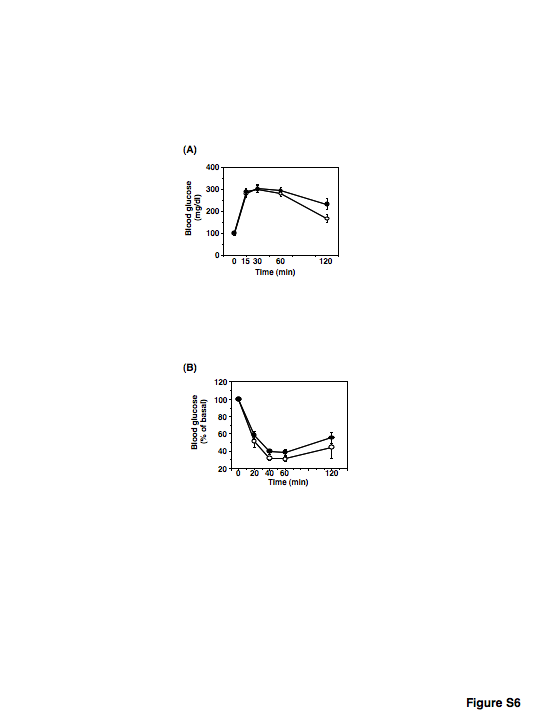

Supplement: Figure S6 — Glucose metabolism in AGRPPdk1−/− and Δ256Foxo1AGRPPdk1 −/− mice. (A) Mean (±SEM) glucose tolerance in AGRPPdk1−/− (open circles) and Δ256Foxo1AGRPPdk1−/− (closed circles) mice at 24 weeks of age: n = 10 mice per genotype. (B) Mean (±SEM) insulin tolerance in AGRPPdk1−/− (open circles) and Δ256Foxo1AGRPPdk1−/− (closed circles) mice at 24 weeks of age: n = 10 mice per genotype. (TIF) [file pone.0018324.s006.tif]

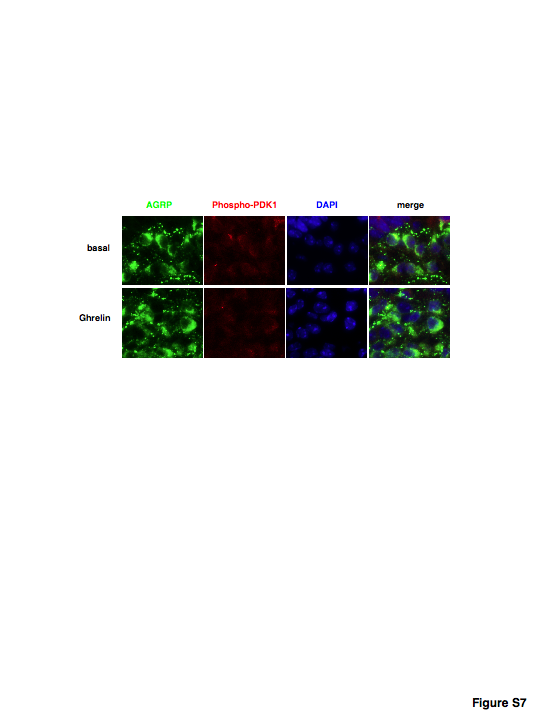

Supplement: Figure S7 — Ghrelin doesn't phosphorylate PDK1 in AGRP neurons. Representative immunofluorescence images of phospho-PDK1 in the hypothalamus of 13-week-old C57bl6 mice after a 24-hour fast (top panel), after an intracerebroventricular injection of ghrelin (bottom panel) (Methods S1). Green, red and blue indicate AGRP, phospho-PDK1, and DAPI staining, respectively. (TIF) [file pone.0018324.s007.tif]
