# Supplementary material for: PDK1-Foxo1 in Agouti-Related Peptide Neurons Regulates Energy Homeostasis by Modulating Food Intake and Energy Expenditure
Source: PLoS One. 2011 Apr 7;6(4):e18324. doi: 10.1371/journal.pone.0018324 (PMC3072380; doi:10.1371/journal.pone.0018324)
Supplement: Methods S1 — (DOC) [file pone.0018324.s008.doc]

**Methods S1**

**Generation of *Δ256Foxo1^aP2^ mice***

The *R26^floxneoΔ256Foxo1^* mice were mated with *aP2Cre* transgenic mice (Jackson Laboratory) to generate *Δ256Foxo1^aP2^* mice.

**Western Blotting.**

Hypothalamic samples were homogenized in buffer containing 50mM Tris-HCl (pH 8.0), 250 mM NaCl, 1% NP40, 0.5% deoxycholate, 0.1% SDS, and protease inhibitors (Roche Diagnostics). After centrifugation to remove insoluble material, lysate samples that contained 50μg of protein were loaded, and proteins were separated on an 8% SDS-PAGE gel, and Western blotting was performed with the indicated antibodies.

**Intracerebroventricular administrations of ghrelin**

Male C57bl/6J mice were studied at 10-13 weeks of age. They were housed individually, and given a normal chow diet and water *ad libitum*. All mice were implanted with a unilateral chronic cannula in the lateral ventricle. Briefly, mice were anesthetized with ketamine (100mg/kg) and xylazine (10mg/kg), and placed in a Kopf stereotaxic frame (Koft Instruments, Tujunga, CA). Then, a double-walled stainless steel cannula was inserted unilaterally into the lateral ventricle, located according to the Franklin and Paxinos atlas. The cannula was anchored firmly to the skull with acrylic dental cement. After 10-14 days of recovery, mice were used for experiments. Ghrelin (200pmol) was injected after a 24-h fast. Then, 30 minutes after injection, mice were deeply anesthetized with sodium-pentobarbitone, and the heart was refluxed from the left ventricle to the right auricle with ice-cold PBS and 4% paraformaldehyde.
